# Gangrene

**Published:** 1892-07-02

**Authors:** 


### THE PRACTITIONER'S MIRROR AND RETROSPECT.

[*The Editor will be glad to receive offers of co-operation and contributions from members of the profession. All letters should be addressed to* The Editor, The Lodge, Porchester Square, London, W.]

### MEDICINE.

### GANGRENE.

In speaking of "gangrene" generally, we mean the death of a circumscribed portion of the body, and its removal in a mass, which is called a "slough." Some of the common causes of gangrene, are injuries, with or without lesion to the main vessels or nerves; strangulations, as in hernia, or in a limb too lightly bandaged; extremes of heat and cold; caustics and certain drugs, as ergot; continued pressure, as in bed sore; diseases of heart and arteries (senile). Gangrene may also occur in many grave diseases. as in diabetes, or in any of the fevers; there is also a special form, which is symmetrical (Reynaud's Disease); and lastly, there is a condition (rarely met with since our hospitals have been so much improved) called Hospital Gangrene. For clinical purposes gangrene is sometimes divided into two kinds, namely, "moist" and "dry"; in the former of these conditions a large portion of living tissue becomes rapidly mortified, there is always considerable inflammation and a free discharge; in the latter condition however, the dead tissue separates slowly from the body, the portion first affected having time to dry up as the disease advances. In moist gangrene there is some obstruction to the return of blood from the part; and in dry gangrene there is always a marked deficiency in the supply of blood to the part. In the majority of cases of gangrene these two forms are usually found together, and sometimes it is impossible to say whether the moist or the dry condition predominates. Having made these few remarks on gangrene as a whole, we will now consider the special features of senile gangrene. Senile gangrene usually commences as the dry form, and is caused by thrombosis in an atheromatous artery, by this means the diameter of the vessel is gradually narrowed, till finally the whole of the blood supply to the part is cut off. There is sometimes a history of some slight injury to the toes or feet, for it always commences in one or both of the lower extremities; the first symptom that is noticed is coldness, which is followed by itching of the part affected—very often the inner side of the great toe; the part then becomes a dusky brown colour, and in a day or two shows a dry black centre; this mortifying process slowly extends day by day, from the toes up to the leg, till the patient dies of exhaustion. Sometimes this condition ceases, and a line of demarcation is formed between the dead and living tissue, and the slough comes away, or is removed by operation. This slow or dry gangrene, if it should reach the leg, always takes on the moist characters when the soft parts forming the calf become implicated; then it is that bullæ appear, inflammation becomes marked, and the disease advances rapidly. Above the gangrenous area the skin is mottled and the superficial veins mapped out. Occasionally in senile gangrene this moist condition is present from the first. When this is so a large area of the extremity becomes rapidly affected, and the course of the gangrene is more active. In the way of treatment, the patient must be kept in bed, with the leg raised, and carefully enveloped in absorbent wool, some iodoform having been dusted over the gangrenous portion; we must avoid placing hot-water bottles too near the affected parts. The nourishment must be given very frequently, in small quantities, and of such a kind as is easily assimilated; stimulants are an absolute necessity; opium must be given, and often in considerable quantities, both to relieve pain and lessen the delirium, which so often ushers in the last stages of the disease. Attention should be directed to the treatment of such constitutional diseases as diabetes, bronchitis, &c.; and we must not forget that fresh air, and if possible, sunshine, are important factors in the proper treatment of these cases. If the gangrene takes on the moist condition, free incisions will be usually required to relieve tension and let out pent-up fluids and gases. The question of amputation of the limb will after a few days come before the surgeon, but the old rule of waiting for a line of demarcation to be formed ought to be very rarely departed from in any form of gangrene, and never in the usual cases of senile gangrene. The only exception to this golden rule is in local gangrene of distinctly traumatic origin (as when due to lesion of a main artery), and it will be wise to operate at once, sufficiently high above the seat of injury to ensure healthy flaps.

### THE POSITION OF THE MEDICAL EXAMINER FOR LIFE ASSURANCE.*—III.

(*Continued.*)

